# Assessment of emotional intelligence in adults with down syndrome: Psychometric properties of the Emotional Quotient Inventory

**DOI:** 10.1371/journal.pone.0236087

**Published:** 2020-07-22

**Authors:** David Sánchez-Teruel, María Auxiliadora Robles-Bello, José Antonio Camacho-Conde

**Affiliations:** 1 Department of Psychology, University of Cordoba, Cordoba, Spain; 2 Department of Psychology, University of Jaen, Jaen, Spain; 3 Department of Medicine, University of Malaga, Malaga, Spain; Universidad Autonoma de Madrid, SPAIN

## Abstract

**Introduction:**

The Emotional Quotient Inventory: Youth version-EQ-i:YV was developed by Bar-On & Parker in 2000 and later translated and adapted for the general Spanish adolescent population by Ferrandiz et al. in 2012. The Spanish scale presents similar psychometric properties to the original version (54 items and five subdimensions). The Emotional Quotient Inventory assesses a set of personal, emotional, and social skills that influence adaptation to and coping with environmental demands and pressures. These factors can influence an adolescent’s success later in life, health, and psychological well-being. Traditionally, research in Down syndrome (DS) has focused on identifying cognitive deficits, relatively little is known about emotional intelligence (EI) and there are no scales that measure EI in people with DS adults.

**Objectives:**

To validate and analyze the psychometric properties of the scale in the clinical population, specifically in Spanish adults with DS (EQ-i: SVDS).

**Methods:**

A cross-sectional investigation was carried out in several stages. Descriptive, exploratory factorial (n = 345), confirmatory (n = 397), and scale reliability analyses were performed with better goodness-of-adjustment indices.

**Results:**

A new scale named Emotional Quotient Inventory: Short Version for DS adults was obtained with a structure of four factors called mood, stress management, interpersonal, and intrapersonal. This new scale was reduced to 25 items. Goodness-of-fit indices were excellent (RMSEA [95% CI] = 02[.01; .03]; CFI = .99; TLI = .98; GFI = .87; AGFI = .89). The internal consistency of the four dimensions and the calculated total score (α = .91, ω = .93 and divided halves = .90) yielded high values in this clinical sample.

**Discussion:**

The results recommend the use of the revised EQ-i: YV, the EQ-i: SVDS, to assess EI in adults with DS. The psychometric properties of this study are satisfactory but have four factors. The findings are discussed in terms of future research and practical implication to gain a more thorough understanding of how this population behaves on both a general and preventive level in order to teach EI properly.

**Conclusions:**

This new version is a valid and reliable tool to evaluate emotional intelligence in people with intellectual disabilities and specifically in Spanish adults with DS.

## Introduction

The concept of emotional intelligence (EI) has generated considerable scientific output and gained significant ground in different fields, including education [1], health [2], well-being and happiness [3–5], and the business world [6–8]. According to Salovey and Mayer [9], EI is an individual’s ability to manage feelings and emotions, discriminate among them, and use this knowledge to direct thoughts and actions. EI includes the ability to accurately perceive, value, and express emotion, the ability to understand emotion and emotional knowledge, and the ability to regulate emotions to promote emotional growth. According to Bar-On [10], the expression of emotional and social intelligence can be used to refer to a set of personal, emotional, and social skills that influence adaptation and coping with the demands and pressures of the environment. This intelligence influences success in life, health, and psychological well-being. Bar-On introduced the expression, Emotional Quotient (EQ), which he would later publish in inventory form. The model of EQ is structured by encompassing different components: (1) intrapersonal; (2) interpersonal; (3) adaptability; (4) stress management; and (5) general mood. Goleman popularized the term but presented it from a new perspective in which the emotional aspect is harmonized with the cognitive one, so EQ encompasses self-awareness, self-management, social awareness, and relationship management.

Goleman [6, 7] spoke of emotional illiteracy in the general population with typical development and in his opinion the Intelligence Quotient (CI) is not a good predictor of success in life; the concept of EI is also needed. If we extend the look of emotional illiteracy to the reality of Down syndrome (DS), as the most frequent form of intellectual disability in the world [11], we see that this group of people is not exempt from emotional distress [12]. There is abundant research examining EI itself in populations with intellectual disability (ID). Studies that have examined concepts related to EI found that individuals with ID (whether with or without DS) exhibit difficulties in domains, such as social cognition and emotional knowledge, even if these researchers did not refer explicitly to the EI term [13–16]. However, these studies focused on people of younger ages.

A central challenge of genetic research in humans is to precisely define phenotypes [17]. The phenotype manifests itself in the DS in a very specific way because the gene product affects different cells, structures, and functions throughout a person’s development. The final phenotypic aspects can be observed clinically in a given individual with trisonomy 21 [18]. In addition, a specific phenotype may be a consequence of a gene but not a direct product of trisomic gene expression [17].

The clinical presentation of DS in each individual is complex and variable, but we can find some common phenotypic features: (1) characteristic facial dysmorphology; (2) a small and hypocellular brain; (3) the histopathology of Alzheimer's disease, which is present in the fourth decade; (4) cognitive impairment to a very variable degree; (5) hypotonia that occurs frequently in newborns; and (6) frequent atypical dermatoglyphic characteristics although the specific subset of these show individual variability [17]. Thus, the behavioral phenotype in SD is not shown rigidly and consistently; it varies from one person to another in its forms and in the intensity of its manifestations and can be used to establish subgroups [19]. Furthermore, rather than one behavioral phenotype, there may be subgroups of children with DS showing different strengths and weaknesses. Thus, it is clear that intellectual disabilities manifest themselves at different levels in people with DS [20].

This phenotype is positively influenced by environmental factors, such as early developmental outcomes, particularly from around the age of two years onwards. These outcomes are the best predictors for performance later in life in children with DS [21, 22]. In addition, several characteristics of a child’s environment and the child itself also influence developmental outcomes although most predictors are not consistent across studies. For example, a higher parental (particularly maternal) educational level is associated with a more favorable developmental outcome in a child [23].

There are several tests that measure EI. The most frequently used include the Trait Meta-Mood Scale (TMMS) that was designed to measure adults’ individual differences in EI [24], depression [8, 25, 26], addiction [27], and stress [28–30]. Tsaousis & Nikolaou [30] found high levels of EI using this test that were associated with good psychological health, whereas other studies linked low EI levels with an increase in depression and anxiety [31].

In the past, some limitations have been raised in screening people with an ID [32] about the validity of self-reported measures in addition to the information provided by their parents and guardians regarding any type of symptomatology. Thus, screening accuracy is questionable [33]. Fortunately, studies involving children born between the 1960s and the late 1980s reported lower performance levels than subsequent studies, including children born in the1990s due to the generalized nature of the early interventions received starting at birth [34, 35]. Thus, there are numerous studies in which these populations are recruited from a more current cohort. These populations have improved cognitive levels and reading and academic skills, suggesting that they may be eligible for anxiety symptom screening using self-reported measures [36–39].

More recently, Herrera and colleagues [40] conducted a study using the Emotional Quotient Inventory: Youth Version (EQ-i:YV; Bar-On & Parker) [41]; their sole objective was to highlight the differences that the instrument reports in Colombian children depending on the setting (rural versus urban), and the results showed that different dimensions of Socio-Emotional Intelligence (SEI) were different according to the setting. Specifically, the results revealed a clear differentiation between rural and urban settings in SEI, obtaining a higher score for urban versus rural children. Esnaola, and colleagues [42] adopted the short version of the questionnaire to analyze developments of different EI dimensions in Spanish adolescents during one academic year and found gender differences only in the stress management sub-dimension. All of these results, some of them based exclusively on exploratory factor analyses [40] and others with a small sample [42], provide interesting information on EI in children and adolescents in the general population who were born after the 1990s. These children and have received adequate schooling, which influences their consistent way of responding to self-reported measures related to emotional analysis.

In DS, the study of socio-emotional competences only recently aroused much research interest versus a study of cognitive and linguistic characteristics [43]. According to Pujol et al. [44] and Flórez, Garvía, and Fernández-Olaria [11], a structural imbalance among the biological regions of the brain is associated with the emotional side (most preserved). A relationship with those related to cognitive and executive functioning (least preserved) is seen in DS. The dialogue or balance between the amygdala and prefrontal cortex governs a good part of the emotional and cognition worlds and executive function. The interconnection between the prefrontal cortex with various nuclei of the amygdala generates a balance of the informational cycles that we receive in both ways (cognition and emotion). Cognition and emotion are protagonists in this bidirectional dialogue between prefrontal-amygdala cortex. The territory loss of one of them is replaced by the other. We cannot understand them in a vision of contrast because there are many interactions between the two. The cognitive and emotional signals are combined through the complex functional networks in the prefrontal cortex. The characterization that defines a person with DS, regardless of their intellectual disability, is their constant reference to their rich emotional world.

Individuals with DS have a variable reduction in intensity in various regions of the prefrontal cortex. This would explain the structural imbalance between the regions associated with the emotional side and those related to the cognitive and executive side for the benefit of the former [11]. In addition, functional resting connectivity through functional magnetic resonance imaging in people with DS is greater in the amygdala and lower in the prefrontal cortex. This translates into greater grassroots activity in the regions involved in cognitive emotional interaction and less in the regions most involved in executive function [44]. If this scenario is indeed the case, then emotional dominance can override any other influence meaning that the resulting behavior (viewed as the person’s overall expression) is marked by emotional influence. Pochon et al. [45] reported only marginal differences in the recognition of different emotions. Two original experimental tasks with similar design were specifically created to examine these differences. The first task included a control task in which six familiar objects were used for this task: (1) a small plastic bottle; (2) a ceramic bowl; (3) a metal cooking pot; (4) a stemmed glass; (5) a plastic citrus juicer; and (6) a plastic kitchen spatula. The second included an emotional task in which six basic emotional facial expressions were presented during this task: (1) happiness; (2) sadness; (3) anger; (4) disgust; (5) surprise; and (6) fear. These tasks had been previously recommended by Moore [46].

A study of the developmental trajectories revealed a developmental difference as the nonverbal reasoning level assessed by Raven’s matrices did not predict success on the experimental tasks in the DS group in contrast to the typical development (TD) group. These results do not corroborate the hypothesis that there is an emotional knowledge deficit in DS and emphasize the importance of using dynamic and strictly nonverbal tasks in populations with language disorders. One might therefore wonder whether the emotional nature of the stimuli makes the task more complicated for adolescents with DS than for TD children. This difficulty explains the greater decline in their scores from one task to the other. This finding raises questions about their abilities to process emotional information and therefore constitutes a limitation in the results of this study. Pochon et al. [45] offered a major methodological advance, which shows that it is possible to effectively evaluate emotional facial expression recognition without using emotional vocabulary, which is essential in DS given the substantial language problems associated with this syndrome [11, 43–45, 47]. It would therefore be relevant to replicate this study using a different matching measure, such as receptive vocabulary levels since vocabulary levels tend to correspond to nonverbal cognitive levels in children with DS.

Cebula et al. [13] described the problems experienced by DS children and adults from different studies. These problems could be related to the specific features noted in studies of social referencing [48]. In children and adolescents with DS, the deficits reported in various studies essentially relate to the recognition of fear, surprise, and anger [49]. The results are contradictory in adults with DS. Hippolyte et al. [50] found problems in recognizing surprise, sadness, and neutral expressions while Carvajal et al. [51] reported no specific difficulties processing emotional expressions. As Cebula et al. [49] indicated, there is still a lack of experimental evidence to conclude that there is a specific emotion-recognition profile characterizing DS. To date, we do not know whether other studies have evaluated EI in DS.

In fact, the ID presents significant intra-syndrome variability [52], which makes it difficult to assess non-cognitive aspects, such as EI, in contrast to other psychological constructs, such as anxiety in adolescents on the autistic spectrum [53]. This fact suggests that it is appropriate to apply indices of goodness of adjustment, which have been methodologically derived from studies based on the general population, to processes of adaptation or psychosocial evaluation in the clinical populations.

Bar-On & Parker [41] developed the EQ-i:YV scale that is designed to measure EI in young people. We used it to measure EI in young adults with DS. They found five factors that assess intrapersonal and interpersonal competencies, stress management, adaptability, and general mood. These data also contain a positive impression subscale designed to detect students who want to give an exaggerated positive impression of themselves. The authors’ responses (in which students come across negatively) and those that reveal social desirability bias (wanting to present oneself in line with how feelings and emotions are socially valued) should be avoided.

There is a short version of Bar-On & Parker’s [41] original EQ-i:YV scale validated by Ferrándiz et al. [54] for use in a young Spanish population. This version describes in detail the validation process and its five factors. This scale provides information on emotional and social competencies of people [41]. This version is effective at evaluating these competencies when it comes to physical and psychological health [55, 56], well-being [57], social interaction [56], academic performance [58], and work-related performance [59]. Interest in the general population focuses on aspects, including academic performance [60], the consumption of psychoactive substances [61], and social and academic adaptation [62]. The scale’s short version has also been validated for use among Hungarian people with the results confirming a 5-factor structure [63]; this scale was also studied in male athletes from Australia aged between 16 and 40 years [64]. Other studies analyzed the youth version and show appropriate psychometric characteristics as it replicates the original factorial structure for different samples consisting of a general population from the United States [65], Lebanon, Peru, and Spain [66]; the latter cohort consisted of gifted and talented students. We also find some preliminary studies involving samples with DS that do not show their psychometric properties [67].

There are no studies on the DS population from this perspective. The current research tested the psychometric properties of the EQ-i:YV in a sample of Spanish adults with DS and compared them with those observed by Ferrándiz et al. [54] who previously validated these values in a typical-population Spanish. More specifically, the study sought to analyze the scale’s structure and internal consistency.

## Materials and methods

### Participants

Spanish adults (55.1% male and 44.9% female) with trisomy 21 (DS; n = 742) between 24 and 32 years old (*M* = 26.04, *SD* = 1.43) participated in this study. The sample consisted of individuals selected from twelve intellectual disability associations in Spain. The presidents and directors of every DS association in Spain [68] were informed about this study both in writing and orally, and 38 positive responses were received: (1) ten from central Spain; (2) twenty from southern Spain; and (3) eight from northern Spain. With the associations’ backing, a letter outlining the study objectives was sent to the parents. Contact was then made with those users whose parents and the participants themselves voluntarily and gladly gave consent for their children to be assessed over two 1.5-h sessions. All families that agreed to participate in this study received a report with the results.

After the application of the tests, the total sample was divided into two groups for structural analysis purposes. All participants were matched for gender and age, but there were more men than women. The most relevant sociodemographic variables are shown in Table 1. The Ethics Committee of the University of Jaen (Spain) approved this investigation.

**Table 1:** Sociodemographic characteristics of adults with Down syndrome.

	Total n(%)	Subsample 1	Subsample 2	*t*	*P*
Gender					
Female	333 (44.9)	141 (42.3)	192 (56.6)	8.23	0.54
Male	409 (55.1)	204 (57.7)	205 (43.4)		
Mean age (SD)	26.04 (1.43)	26.1 (1.60)	25.9 (1.2)	2.56	0.93
TOTAL	742	345	397		

*t* = Between-group test statistic

All participants had an intelligence quotient (IQ) >50 (Table 2). According to the World Health Organization (WHO), these participants are classified as having mild intellectual disabilities [69]. Their IQs were assessed using the Kaufman Brief Intelligence Test (K-BIT; Kaufman & Kaufman [70]), which is an assessment tool designed for individuals aged 4 to 90 that measures all areas of general intelligence. It contains 82 items and consists of subtests: (1) verbal (45 vocabulary-based items) and (2) non-verbal (37 matrices items). Cronbach’s alpha and test–retest coefficients in this test were adequate.

**Table 2:** Descriptive statistics obtained from the Kaufman Brief Intelligence Test (K-BIT) in study participants (n = 742).

K-BIT	M	SD	Min.	Max.	r_xx_	Α	S.	K.
							SE (.09)	SE (.18)
Vocabulary	52.22	1.32	49	56	0.63	0.60	–0.31	–0.26
Matrices	61.96	1.46	59	62	0.67	0.67	–0.15	–0.02
TOTAL	54.53	1.01	51	55	0.69	0.68	0.10	–0.08

M = Mean; SD = Standard deviation; Min = Minimum value; Max = Maximum value; S = Skewness; K = Kurtosis; SE = Standard error of skewness and kurtosis; rxx = Split halves; α = Cronbach’s alpha

### Measure

The EQ-i:YV questionnaire [41] was validated for use among Spanish adolescents by Ferrándiz et al. [54]. It is based on the 60-item adult version that assesses several EI dimensions on a 4-point Likert scale ranging from 1 (“It never happens to me”) to 4 (“It always happens to me”). The EQ-i:YV in Spanish measures Emotional-Social Intelligence (ESI) after considering five components: (1) intrapersonal (INTRA); (2) interpersonal (INTER); (3) adaptability (A); (4) stress management (SM); and (5) general mood (GM). The INTRA subdimension measures emotional understanding or the ability to express and communicate one’s feelings and needs. INTER refers to the ability to listen, understand, and appreciate the feelings of others. The subdimension A measures the ability to deal with everyday problems. SM refers to the control we have in maintaining peace of mind and coping with stressful situations, and the GM subdimension measures optimism and the ability to maintain a positive appearance and joy. The English version contains a “Positive Impression” subdimension (6 items) that is not included in the Spanish version. The version adapted by Ferrándiz et al. [54] for use among Spanish young adults in the general population was chosen for this study.

### Procedure

The first steps of this study were taken after obtaining all relevant permissions from the participating associations and the verbal commitment by psychologists from these associations to collaborate. First, before the application of the tests, the second author provided a virtual training session through Skype for 1.5 h to the psychologists of the participating associations to help (if necessary) adults with DS. Second, the tests were executed by the adults themselves who gave their written consent to participate. Some of the adults with DS who presented difficulties were helped by the collaborating psychologists; however, such assistance was rare. Finally, the collaborating psychologists sent all of the evaluation booklets to the first author of this manuscript by e-mail and by post.

### Data analysis

This instrumental study was conducted in several stages. First, missing data accounted for less than 2% for all variables, and a multiple imputation method (SPSS) was used to impute missing values [71]. Second, internal consistency testing, item analysis, and exploratory factor analysis (EFA) of the subscales covered in a subsample were performed. The FACTOR program [72] was used for the EFA. This program is suitable for the exploration of ordinal data and offers the possibility of calculating the proportion of the shared variance explained for each one of the extracted factors [73]. For the EFA, the selected factor extraction procedure was the Minimum Rank Factor Analysis (MRFA) [74] using the parallel analysis (PA) with polychoric correlations [75] with optimal implementation [74] for the dimensionality assessment of ordinal-level data. The scale’s original factor structure (five subdimensions) was maintained for performing analyses. Regarding the rotation procedure used to obtain maximum parsimony when interpreting the factor solution, a classical method of oblique rotation was used, specifically the direct oblimin rotation method with a delta value equal to 0 [76].

A parallel analysis (PA) [77] was subsequently applied using the SPSS macro developed by O'Connor [78]. In the parallel analysis, 100 sets of random data with the same number of variables and observations as the actual dataset were created. For a factor to be retained, the eigenvalue for the actual dataset had to exceed 95% of the random matrix eigenvalues for the same factor [79]. Third, a factorial confirmation analysis (CFA) with SPSS AMOS was performed on another subsample of adults with DS to determine whether the structure obtained with previous analyses was confirmed. The method used in the confirmatory analysis was unweighted least squares (ULS) with bootstrap procedures (given multivariate non-normality) [80]. The fit indices used were the χ2/df ratio, root mean square error of approximation (RMSEA), comparative fit index (CFI), Tucker-Lewis Index (TLI), and adjusted goodness-of-fit index (AGFI), and gamma index (GI). The goodness-of-fit model is deemed satisfactory if TLI and CFI ≥ 0.95 and the RMSEA is close to 0.06 [81].

## Results

### Descriptive statistics and item analysis (n_1_ = 345)

In general, the data offered by the analysis of items and internal consistency showed an important variability in asymmetry and kurtosis in this sample. This indicates the non-univariate normality of this sample [82]. Cronbach's alpha of the GM subdimension was 0.53, the alpha of the SM subdimension was 0.61, the alpha of the A subdimension was 0.46, the alpha of the INTER subdimension was 0.62, and the INTRA subdimension was 0.59. In the item total and alpha correlation, there were some problematic items in the GM subdimension (items 9, 29, and 40), in the SM (items 3 and 6), in A (items 12, 30, 34, 38 and 44), in INTER (item 2), and in INTRA (item 7). In particular, the correlation of the items with the total scale presented very low scores (<0.30). The Cronbach’s alpha increased if items with low item-total scale correlations were eliminated. However, it was decided to keep these items to assess their actual impact in subsequent factor analyses [83, 84] as shown in Table 3.

**Table 3:** Descriptive statistics, skewness, and kurtosis indices: Item analysis of the EQ-i:YV.

EQ-i:YV	*K-S*	S	K	*r item-total*	*α if item deleted*
		*SE (-*.*01)*	*SE(2*.*69)*		
Item 1 (GM)	.90**	.17	.72	.67	.45
Item 4 (GM)	.81**	–.14	–1.11	.49	.48
Item 9 (GM)	.80**	.03	–.77	.02	.81
Item 13 (GM)	.86**	–.05	1.01	.31	.48
Item 19 (GM)	.89**	–.09	–.92	.35	.50
Item 23 (GM)	.80**	.05	–.86	.41	.52
Item 29 (GM)	.87**	.09	–.81	.21	.87
Item 32 (GM)	.86**	.07	–.78	.72	.52
Item 37 (GM)	.90**	.13	–.88	.35	.50
Item 40 (GM)	.67*	.49	–.91	.19	.96
Item 47 (GM)	.70**	–.37	–.26	.59	.50
Item 50 (GM)	.83**	.49	–.61	.72	.52
Item 56 (GM)	.88**	–.42	–1.02	.74	.51
Item 60 (GM)	.75**	.56	–.64	.48	.49
Item 3 (SM)	.00**	–.01	1.90	.13	.89
Item 6 (SM)	.90**	–.08	–.97	.11	.79
Item 11 (SM)	.90**	.07	-.83	.59	.50
Item 15 (SM)	.80**	.49	–.61	.72	.52
Item 21 (SM)	.86**	.20	-1.02	.48	.49
Item 26 (SM)	.83**	–.06	–.89	–38	.51
Item 35 (SM)	.87**	–.04	–1.28	.85	.50
Item 39 (SM)	.89**	–.16	–.67	.42	.46
Item 46 (SM)	.90**	.28	–.22	.66	.52
Item 49 (SM)	.67*	–1.95	2.56	.55	.73
Item 54 (SM)	.70**	–2.61	2.25	.71	.66
Item 58 (SM)	.83**	–.95	.55	.47	.54
Item 12 (A)	.87**	.10	–1.12	.15	.79
Item 16 (A)	.91**	-.03	-.92	.35	.56
Item 22 (A)	.97**	.06	–.78	.45	.53
Item 25 (A)	.55**	–.10	–1.07	.32	.52
Item 30 (A)	.87**	.18	–1.01	.17	.80
Item 34 (A)	1.27*	.08	–1.11	.11	.77
Item 38 (A)	.81**	3.32	–3.47	.20	.69
Item 44 (A)	.38**	–.33	–.82	.19	.73
Item 48 (A)	1.35**	–.42	–1.29	.40	.52
Item 57 (A)	.17**	–.41	–.48	.42	.54
Item 2 (INTER)	.18**	.22	–.93	.11	.79
Item 5 (INTER)	.25*	–.18	–.46	.31	.53
Item 10 (INTER)	.25**	–.04	–.78	.50	.51
Item 14 (INTER)	.32**	.01	–.43	.41	.51
Item 20 (INTER)	.26**	.16	–1.04	.54	.53
Item 24 (INTER)	.25**	–.18	–.46	.78	.48
Item 36 (INTER)	.14**	.28	–1.12	.70	.53
Item 41 (INTER)	.27**	–.36	–.94	.45	.53
Item 45 (INTER)	.28**	–.36	–.91	.35	.56
Item 51 (INTER)	.26**	–.82	1.14	.32	.52
Item 55 (INTER)	.16**	–.32	–.86	.43	.49
Item 59 (INTER)	.24**	.28	–1.09	.62	.52
Item 7 (INTRA)	.90**	–.12	–1.17	.17	.83
Item 17 (INTRA)	.81**	–.30	–.23	.36	.50
Item 28 (INTRA)	1.29*	3.43	–4.04	.47	.54
Item 31 (INTRA)	.91**	.28	–.45	.31	.53
Item 43 (INTRA)	.86**	.10	–.81	.52	.51
Item 53 (INTRA)	.86**	–.24	–1.20	.49	.48
TOTAL	.98**	.05	1.68	1	.57

S = Skewness; K = Kurtosis; SE = Standard error of skewness and kurtosis; K-S = Kolmogorov–Smirnov test

*Significant correlation at the 0.05 level (bilateral)

**Significant correlation at the 0.01 level (bilateral); GM = General Mood SM = Stress Management; A = Adaptability; Inter = Interpersonal; and Intra = Intrapersonal.

### Exploratory factor analysis (n_1_ = 345)

The Kaiser–Meyer–Olkin measure of sampling adequacy index (KMO = 0.89), Bartlett’s test of sphericity (χ^2^ = 2146.2; p <0.001), and the determinant of the correlation matrix (0.005) showed data suitability for factor analysis [78]. The FACTOR program compares the mean or the 95^th^ percentile of the factor’s percentage of common variance explained from the randomly permutated data to the observed explained common variance from the sample [72]. If a factor’s observed percentage exceeds the random percentage, the factor is then retained. This process occurred five times in the case of the EQ-i:YV. Therefore, five dimensions were extracted via exploratory factor analysis, which explained 22.20% (Factor I), 19.23% (Factor II), 16.27% (Factor III), 14.67% (Factor IV), and 11.50% (Factor V) of the variance (based on eigenvalues) as shown in Table 4. Factor 1 (GM) brought together 14 items with a single factor saturation of ≥0.48. Factor 2 (SM) produced a further 12 items with a factor saturation of ≥0.55. Factor 3 (A) gave another 10 items with a factor saturation of ≥0.31. Factor 4 (INTER) brought together 12 items with a factor saturation of ≥0.44. Finally, Factor 5 (INTRA) produced six more items with a factor saturation of ≥0.49. However, not all items load onto their theoretical dimension leading to several complex items with crossed loads medium and large. It is therefore decided a parallel analysis (PA) [77].

**Table 4:** Emotional Quotient Inventory: Youth Version (EQ-i:YV) exploratory factor analysis in the subsample with Down syndrome (DS) with n_1_ = 345.

EQ-i:YV	*Dimensions*					
	1	2	3	4	5	h^2^
GM						
Item 1	**.53**	–.11	.14	.19	.01	.58
Item 4	**.63**	**.59**	.27	.09	.16	.43
Item 9	**.38**	**.45**	**.67**	.23	.11	.38
Item 13	**.49**	**.40**	.08	.21	.32	.39
Item 19	**.48**	.07	**.30**	.25	.13	.48
Item 29	**.39**	.06	**.45**	.16	.04	.53
Item 23	**.55**	**.54**	.06	.28	.29	.83
Item 32	**.63**	.16	.21	.05	.16	.53
Item 37	**.62**	.09	.17	.11	.04	.89
Item 40	.23	**.41**	**.32**	.26	.09	.12
Item 47	**.52**	.23	.25	.03	.19	.52
Item 50	**.59**	.11	.02	.18	.51	.71
Item 56	**.56**	.12	.17	.09	.19	.54
Item 60	**.72**	.10	.19	.21	.01	.63
SM						
Item 3	**.36**	.12	.29	.19	.11	.19
Item 6	**.45**	**.34**	**.42**	.07	.21	.26
Item 11	.09	**.54**	.12	**.32**	.17	.52
Item 15	-.13	**.59**	.29	.21	.02	.54
Item 21	.15	**.57**	.04	**.30**	.18	.48
Item 26	.02	**.56**	.07	.15	**.32**	.60
Item 35	.15	**.89**	.17	.06	.14	.72
Item 39	.06	**.77**	.23	.01	.18	.85
Item 46	.12	**.55**	.24	.19	.05	.52
Item 49	.10	**.90**	.02	.04	.22	.91
Item 54	.24	**.71**	.21	.02	.13	.49
Item 58	.14	**.82**	.11	.02	.23	.51
A						
Item 12	.21	**.66**	**.53**	.19	.13	.22
Item 16	**.39**	.10	**.66**	.16	**.32**	.97
Item 22	**.34**	.18	**.55**	.01	-.11	.21
Item 25	.22	.18	**.91**	.11	.23	.62
Item 30	.19	**.42**	**.38**	.26	.16	.41
Item 34	.29	**.45**	**.36**	.11	.26	.32
Item 38	**.33**	.28	**.31**	.19	.10	.16
Item 44	**.56**	**.31**	**.48**	.03	.24	.11
Item 48	.03	.17	**.79**	**.39**	.12	.75
Item 57	**.35**	.06	**.59**	.13	.23	.67
INTER						
Item 2	**.46**	.24	**.31**	**.39**	.26	.12
Item 5	.11	.25	.14	**.96**	.24	.15
Item 10	.07	.15	.26	**.79**	.14	.54
Item 14	.07	.14	.06	**.55**	**.37**	.77
Item 20	.01	.13	.12	**.73**	.19	.39
Item 24	.10	.28	.18	**.91**	.07	.98
Item 36	.04	.12	.09	**.58**	.19	.83
Item 41	.01	**.32**	.20	**.52**	**.51**	.73
Item 45	.13	.02	.13	**.67**	**.41**	.61
Item 51	.03	–.16	.18	**.95**	–.12	.21
Item 55	.12	–.11	.13	**.64**	.04	.97
Item 59	.10	.11	.05	**.75**	**.62**	.88
INTRA						
Item 7	**.35**	.29	.23	.17	**.39**	.35
Item 17	.21	.15	.06	.13	**.61**	.44
Item 28	.19	.11	.09	.14	**.96**	.27
Item 31	.12	.07	.13	.26	**.66**	.91
Item 43	.21	.11	.08	–.19	**.72**	.97
Item 53	.06	.17	.12	.09	**.51**	.76
% variance	22.20	19.23	16.27	14.67	11.50	

Rotated loading with values >0.30 in bold type; h^2^ = Commonalities; GM = General Mood; SM = Stress Management; A = Adaptability; Inter = Interpersonal; and Intra = Intrapersonal.

In the second stage, all complex items with medium and high factorial loads in several subdimensions were eliminated. The parallel analysis (PA) [77] using 95th percentile eigenvalues indicated that a factorial solution of four subdimensions would be appropriate. A second factor analysis was performed using the four subdimensions that subjected to an oblimin rotation. Cumulatively, the four factors explained 42.12% of the overall variance. In order to interpret the contribution of each item to the factor, a minimum 0.30 factor-loading criterion was used (see Table 5).

**Table 5:** Psychometric properties and exploratory analysis of the EQ-i:SVSD (n_1_ = 345).

			*Dimensions*			
	M	SD	1	2	3	4
MD						
Item 1	2.50	1.43	**.98**	.15	.18	.03
Item 32	2.03	1.29	**.45**	.13	.09	–.46
Item 37	1.28	.04	**.62**	–.07	.14	.18
Item 47	2.50	1.32	**.53**	–.15	.26	.20
Item 50	1.70	1.80	**.69**	.09	-.18	.13
Item 56	1.54	1.99	**.56**	.10	.17	.05
Item 60	.94	.73	**.82**	.11	.21	.03
SM						
Item 3	1.60	.28	.16	**.66**	.23	–.09
Item 15	2.52	1.84	–.14	**.56**	–.01	.17
Item 35	1.63	.39	.10	**.79**	.12	–.03
Item 39	2.99	1.45	.05	**.51**	.02	.19
Item 46	1.96	1.44	.11	**.53**	.16	.23
Item 49	1.88	1.73	.03	**.92**	.08	.21
Item 54	2.81	.56	.22	**.88**	.14	.02
Item 58	0.73	.78	–.03	**.73**	.06	.19
INTER						
Item 5	1.48	1.13	.16	.18	**.99**	–.07
Item 10	2.59	1.35	.19	.13	**.23**	.16
Item 20	1.83	1.88	–.03	.09	**.78**	.04
Item 24	2.56	1.30	.11	.16	**.74**	.13
Item 36	2.67	1.67	.16	–.21	**.56**	.16
Item 51	1.98	1.39	.21	–.23	**.49**	.22
Item 55	2.78	1.71	.06	.12	**.81**	.20
INTRA						
Item 17	2.87	.83	.12	–.02	.12	**.98**
Item 28	1.99	.77	.22	–.15	.20	**.66**
Item 31	1.18	1.10	-.06	.13	–.05	**.47**
Item 43	2.07	1.88	.18	.26	.11	**.91**
Item 53	2.54	1.56	.23	.08	–.22	**.77**
Eigenvalue			4.60	3.19	2.76	2.21
% variance			31.34%	22.10%	19.32%	16.59%

All Loadings in bold (minimum .30) are significant; MD = Mood; SM = Stress Management; Inter = Interpersonal; and Intra = Intrapersonal.

### Confirmatory factor analysis (n_2_ = 397)

**Fig 1:**
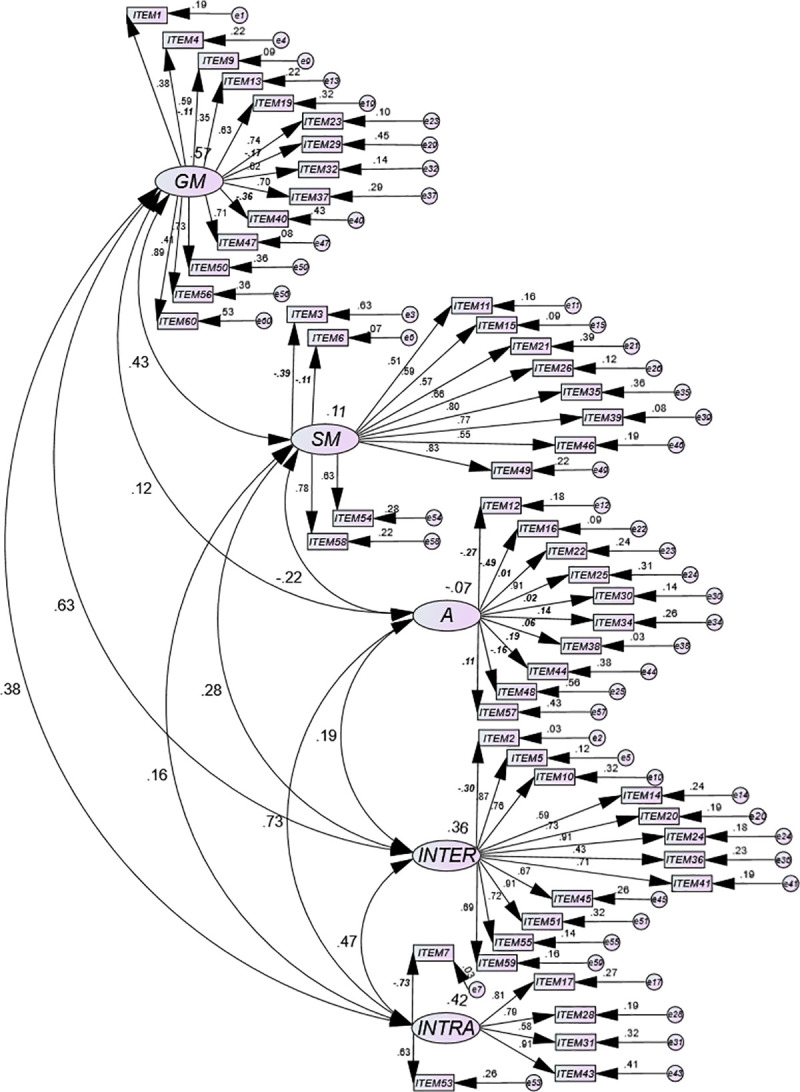
Path diagram of the five-dimensional model corresponding to the Emotional Quotient Inventory: Youth Version (EQ-i:YV) in adults with Down syndrome (DS). Model 1 = full scale.

The results garnered from the univariate and multivariate normality analysis in the second sample of adults with DS (n_2_ = 397) showed that there was neither univariate nor multivariate normality in item distribution (Mardia = 437.51) [85]. Fig 1 shows a path diagram of the EQ-i:YV results for adults with DS (Model 1 = full scale). The standardized weight values (coefficients from β) were negative and very low for a large number of items in various subdimensions (marked in bold and italics in the figure), specifically within the general mood (GM) –0.11 in item 9, –0.17 in item 29, and –0.36 in item 40. The same findings existed in the A subdimension in items 12 (–0.27), 16 (–0.49), 22 (0.01), 30 (0.02), 34 (0.14), 38 (0.06), 44 (0.19), 48 (–0.16), and 57 (–0.11). This pattern was observed in the interpersonal subdimension (INTER) item 2 (−0.30) and in intrapersonal (INTRA) item 7 (−0.73). In addition, negative covariances are detected between SM and A (–0.22) or very low such as those observed between the GM and A (0.12) or between the subdimension SM and INTRA (0.16).

**Fig 2:**
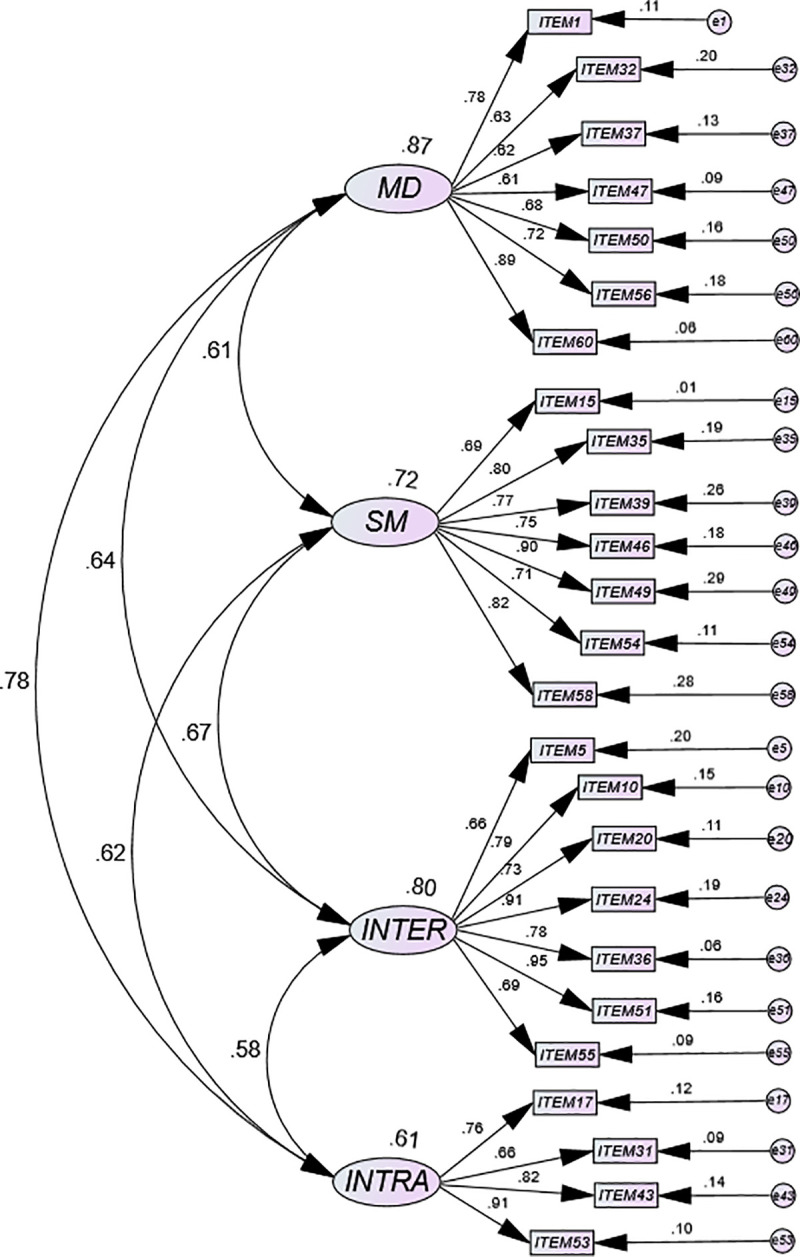
Path diagram of the four-dimensional model corresponding to the termed as EQ-i:SVDS (Model 2).

Fig 2 shows high factor loads (≥0.60) for most items in the EQ-i:SVDS path diagram of Model 2. Standardized weight values (coefficients from β) ranged from 0.61 for item 47 (GM) to 0.95 for item 36 (INTER). In addition, the greatest covariances occur between SM and INTER subdimensions (0.78) in addition to between GM and INTRA (0.67) and INTER subdimensions (0.64).

Table 6 shows that Model 2 (with 25 items), unlike Model 1 (with 54 items), produced very good indexes of goodness-of-fit of EQ-i:YV in this population. Specifically, Model 1 presents important differences between the CFI and TLI indices and a higher score of 0.05 for the RMR. However, Model 2 has an adequate and significant χ2/df. All remaining indices were excellent with an RMSEA value (95% confidence interval [CI]) < 0.06, adequate scores for CFI and TLI, and GFI and AGFI values>0.85 limit with a high agreement among the evaluated goodness-of-fit indices. Based on these results, the acceptability and goodness-of-fit of model 2 was considered strong.

**Table 6:** Goodness-of-fit indices for the EQ-i:YV in the DS subsample (n_2_ = 397).

	χ^2^	df	χ^2^/df	*p*	RMSEA (95% CI)	CFI	TLI	RMR	GFI	AGFI
Model 1	128.12	58	2.21	.00	.06 [.01; .08]	.97	.86	.08	.79	.81
Model 2	122.45	58	2.11	.00	.02 [.01; .03]	.99	.98	.03	.87	.89

Model 1 = Exploratory factorial analysis of the 54 items (EQ-i:YV complete); Model 2 = Exploratory factorial analysis of the 25 items (EQ-i:SVDS) *χ*^*2*^ = Chi-square; df = degrees of freedom, *χ*^*2*^*/df* = Chi-square goodness-of-fit index; p = significance level; RMSEA = Root mean square error of approximation; CFI = Comparative Fit Index; TLI = Tucker-Lewis Index; RMR = Root mean residual (similar to the RMSR for Factor 10.3); GFI = Gamma index; and AGFI = Adjusted goodness-of-fit index.

The results referring to the normality of the short scale (Model 2 = 25 item) showed that the distribution of the measuring variables (EQ-i:SVDS) did not present univariate normality. This finding is supported by data obtained through the skewness and kurtosis indices that yielded extreme scores (− 2; 2) as shown in Table 7. Furthermore, the results corresponding to the internal consistency (alpha, omega, and split halves) of all four dimensions and the EQ-i:SVDS’s total calculated score yielded high alphas and omegas across all dimensions and in the total score. Finally, the split-half reliability procedure for each dimension, as well as for the reduced scale (model 2 = 25 items), gave equally adequate coefficients (Table 7).

**Table 7:** Variable descriptive for the adults with DS (Model 2).

	M	SD	Min.	Max.	K-S	*p*	r_xx_	ω	α	S.	K.
										SE (.32)	SE(.63)
EQ-i:SVDS	102.09	9.11	74	130	.16	.00	.90	.93	.91	–1.11	2.31
Mood	43.12	5.32	11	29	.54	.00	.88	.89	.86	.27	.44
Stress management	39.16	1.27	24	48	.19	.00	.79	.83	.79	–1.26	1.29
Interpersonal	33.22	3.16	39	43	.41	.00	.86	.88	.83	.40	.51
Intrapersonal	28.16	1.22	22	33	.28	.00	.81	.86	.80	–1.97	2.11

EQ-i:SVDS = Emotional Quotient Inventory: Short Version for Down Syndrome; M = Mean; SD = Standard deviation; Min = Minimum value; Max = Maximum value, K-S = Kolmogorov–Smirnov test; p = Significance level; r_xx_ = split halves; ω = Omega coefficient; α = Cronbach’s alpha; S = Skewness; K = Kurtosis; and SE = Standard error of skewness and kurtosis

## Discussion

This study provided preliminary psychometric support for the use of the EQ-i:YV [41, 54] as a research measure to assess EI in adults with DS. This new brief scale is useful and necessary for working with this population because it is not a normal population that can be analyzed using normal criteria; it is a population considered to be within the intellectual disability group and needs a scale adapted to their special needs. Therefore, it would be useful to use this scale for population with similar characteristics to those in the sample. This scale will have an impact on the group with DS because it is a tool that provides the evaluation of the development of emotional competences with the conviction that these can also be enhanced according to the results achieved in the assessment. It can help to incorporate this tool in the school evaluation of children with DS—it is already more urgent to enhance emotional intelligence than cognitive intelligence despite the insistence of teachers in the latter [12].

The new version of the EQ-i:YV scale in adults with DS with four factors was not similar to the one reported by Ferrándiz et al. [54], as the resulting scale was shorter and with fewer subdimensions. The factors of the new version, termed the Emotional Quotient Inventory: Short Version for Down Syndrome (EQ-i:SVDS) were labeled as Factor I- MD, Factor II-SM, Factor III-INTER and Factor IV-INTRA. The INTRA subdimensions of the scale measures an individual’s ability to solve problems (ability to identify, define, create, and implement possible solutions), the ability to validate one's own emotions and distinguish the real from the real, and the flexibility or ability to adjust one's own emotions, thoughts, and behaviors to changing situations and conditions. Item analysis and EFA show the existence of adequate functioning of most items in this sample. However, through PA, some complex elements with high factorial loads (>0.30) were detected not only in their theoretical dimension and resulted in four subdimensions (rather than five). Some of the measured emotional abilities include a weak point in the behavioral phenotype of adults with DS [11], that is, the effects of DS in the areas or lobes of the prefrontal cortex (dorsolateral and cingulate) and in the contact routes with various subcortical structures, such as the limbic system are not strong. The modified scale lists only some of the possibly affected areas that will influence an individual’s ability to understand and control emotional life [12]. Although this population exhibits a special type of emotional sensitivity, that is to say, this population exhibits a preference for emotional information of a positive nature and elimination of that of a negative nature [11, 86, 87], this scale shows clear limitations in interpreting, categorizing, and defining specific emotions within this population. Emotional sensitivity is due to the person’s state since the value of the mean score for each item is within the intermediate values of the scale (2–3) with the items belonging to INTER and GM obtaining higher scores, while items belonging to INTRA and SM obtained the lowest scores. In addition, the data show a decrease in scores for the SM, A, and MD sub-dimensions and the total EI as a student’s age increases, which could indicate that as students grow older they perceive themselves as less emotional and more socially competent in these dimensions [54]. It seems incoherent for a cognitive construct focused on emotional information processing, such as EI, to be represented by self-perceived general mood as any type of intelligence should be demonstrated by intellectual performance. However, it could be possible that a person who presents a better mood is precisely one who is able to self-manage it as consequence of a better and more effective perception, expression, facilitation, and emotion management [54]. This person’s emotional vocabulary is limited, and this individual easily confuses indirect requests, suggestions, and insinuations [12, 88]. In this type of population, there seems to be a response pattern tending to positive emotions (happiness, joy) and this type of response manifests itself in situations in which people with DS are not sure of the correct answer [12, 89]. Regarding the SEI dimensions, it is important to notice the relevance of the first factor (GM) within the scale as this factor consists of the greatest number of items. Bar-On also places this factor at the first level and defines it as the ability to enjoy life by integrating both happiness and optimism. Also, this is an essential element in interactions with others; this attribute is a motivational component in problem solving and stress tolerance [41, 90]. The value of the mean score for each item is within the intermediate values of the scale (2–3) with the items belonging to INTER and GM being the ones that obtained higher scores, while items belonging to INTRA and SM received the lowest scores. In quantitative terms, in a study by Ferrándiz et al. [54], the dispersion analysis showed that the subjects of the sample chose all of the 4-point response ranges on every inventory item. This dispersion is a positive factor regarding the adequacy of the inventory in this sample; thus, the majority of items have a mean of around 2 to 3 points. Also, the internal consistency analysis of the items showed positive values for the scale validity in this sample. In addition, the Cronbach’s alpha coefficients of the five dimensions of SEI (between 0.63 and 0.80) were within the required values in terms of psychological test validity.

We next decided to evaluate two models through a CFA in another sample of adults with DS: Model 1 where the structure of the scale was the same as the adaptation to Spanish in the general population [54] and Model 2 where complex items with crossed loads were eliminated. These results show that the A (Adaptability) factor is the one that loses the most items specifically 9 out of 10 that make up this subdimension. Factor A is particularly affected, which is not surprising if we remember that this factor evaluates the ability to deal with everyday problems as well as the ability to identify, define, and generate and implement possible solutions. It includes discerning between the experienced and the true as well as the ability to adjust to emotions, thoughts, and behaviors when situations and conditions change (behavioral flexibility), i.e., the weak points at the phenotype level of DS [13, 50]. The results confirmed that the test works better for this group of people with DS without this factor.

In this sense, people with DS have cognitive disabilities because their limitations focus exclusively on the aspects of reasoning, abstraction, and conceptualization. Their limitations are not in an emotional and volitional contents. They understand what they want both in the emotional sense of loving, having affection or inclination towards someone or something, and in their sense of wanting and having will or interest in something [43, 44]. Hence, taking acceptance to the extreme, we could go so far as to say that people with DS have cognitive but not mental disabilities in all the extension of the term because the mind has two other components—the will and the affections—in which it is yet to be demonstrated that people with DS have disabilities [11, 12, 88]. This work tries to add research in this sense, i.e., we must broaden the view of emotional illiteracy by extending it to the reality of DS. These people are not exempt from emotional distress. Marginalization and the problems of social exclusion that they live from an early age at the school and the social level. They can have attention and reasoning difficulties as well as anxiety and depression. This situation is aggravated by the inherent limitations of intellectual disability.

In the EQ-i:YV path diagram of Model 2 (eliminating the factor A and those with cross loads in other subdimensions), all items correlated with the corresponding dimension meaning that the scale is valid. This new scale (with 25 items), unlike the original scale (with 54 items), produced very good indexes of goodness-of-fit in this population. All subdimensions of the new 25-item scale (EQ-i:SVDS) are made up of seven items except for the intrapersonal subdimension [3]. The highest weights correspond to the latent variables Mood-MD (.87) and Interpersonal-INTER (.80). This was the expected outcome if we remember the ability to enjoy life coupled with a happy disposition and an optimistic outlook that characterize people with DS [11, 12, 88]. The testimonies of parents and siblings (having overcome their initial disconcerted reaction) generally reflect shared opinions, highlighting the kindness, friendliness, and affection shown by their DS children, brothers or sisters, at least during the early years of life [12, 88]. These aforementioned issues—together with the optimism that falls outside their intellectual disability—seems to demonstrate that the group enjoys a rich emotional world; this is the opposite of what other authors have observed in their experiments [51].

The reliability of the EQ-i:SVDS was measured through three procedures (Cronbach's α, omega, and two halves). The results show an adequate degree or accuracy to measure emotional intelligence in this clinical sample of people with DS. Specifically, the reduced scale of 25 items have high indices of consistency (α = .91 and Ω = .93), and the subdimension that presents the most adequate is Mood-MD (α = .86; Ω = .89); the lowest is SM (α = .79; Ω = .83). The same is true for the two-half procedure (MD = .84; SM = .79). In Ferrándiz et al.'s [54] validation study on Spanish adolescents, the reliability indices were between .63 for INTRA and .80 for GM (referred to as MD in this short version).

On the other hand, the CFA in Model 1 (full scale) needs to be discussed. The fact that the two goodness-of-fit indices (CFI and TLI) differ indicates a clear lack of specification of dimensionality in the proposed model. However, it is interesting to note that the TLI (not so much the CFI) is modulated by the instrument items number (54 item) and by the sample size (in this case medium n = 397) [91]. These aspects could add discrepancy between these indices of goodness-of-adjustment (CFI/TLI).

This is why it is important to adapt evaluation instruments in clinical samples. This is not only seen in university students and the general population, but it is also essential that these instruments be brief. Thus, the two confirmatory analyses performed (model 1 and model 2) led to important improvements in the structural validity of the scale. This eliminates this discrepancy in the goodness-of-fit indexes (CFI and TLI) and also verifies that the differences between CFI and TLI in model 1 were due to the inclusion of items not suitable for this clinical population of adults with DS and with high and medium factorial loads in several subdimensions. Clearly, new theory and research is needed to develop and/or adapt existing adapting evaluation tools for adults with DS [92].

The validation process behind self-report questionnaires intended for people with intellectual disabilities has notable methodological limitations [33]. Despite this, it is necessary to understand the characteristics of these populations themselves and not to adhere to standard practice, i.e., comparing them with typically developing populations—such an approach prevents us from fully appreciating the possibilities and variability shown by the adults with DS that we study. In the past, we have attempted to address these limitations by using parents as informers. However, in the current climate, many young people learn to read and write in an almost standardized manner. In fact, together with intra-syndrome variability [93], we find ourselves able to access those described in this sample as has already been done with other population types, e.g., when exploring other constructs such as anxiety in adolescents with autism spectrum disorders [32]. Furthermore, our study is limited in that it only included adults with a mild intellectual disability (MID); many adults with DS have moderate or severe disabilities. Therefore, our results may not be generalizable to the entire DS population.

Another limitation worth highlighting is not being able to study convergent validity by comparing the EQ-i:YV with other scales that also assess EI. In our sample, this validity has not been established since of all the scales used, the EQ-i:YV is the only one whose results we have managed to use coherently. Furthermore, we consider the fact that the special kind of emotional sensitivity shown by our participating adults with DS may have been concealed by difficulties in verbally expressing themselves and their lesser ability to take the initiative and voice their preferences.

The results have shown that the adaptation of the EQ-i:YV to adults with DS is a reliable, valid, and, above all, short tool for measuring EI in this population. The psychometric properties of this study are satisfactory and confirm the four-factor structure. These results mean that it is possible to use the EQ-i:YV to assess EI in adults with DS. This would allow us to gain a more thorough understanding of how this population behaves on both a general and preventive level in order to teach EI properly. It is especially relevant in a clinical setting where emotional distress signs and suspicions arise.

In addition, the cross-sectional nature of the study precludes any conclusions about the direction of the relationship between emotional intelligence of people with DS and their well-being or difficulties. Longitudinal studies are essential to monitor the fluctuations in emotional intelligence over an extended period, and to provide a broader picture of emotional intelligence development from childhood to adolescence into adulthood.

Also, this research was conducted in a Spanish context in which adults to are exposed to an educational system that is traditionally anchored in the idea of the educational inclusion of people with intellectual disabilities. In addition, it is a group of parents of people with DS who have historically worked in this country to benefit these people so that they can integrate into society as people with full rights [11, 12]. This may affect different aspects of their mental health and emotional well-being.

Finally, this type of research study aimed at specific populations is important when it comes to making social, educational, and psychological advances in developing emotional learning programs consistent with discoveries made on how to grow EI in adults with DS as raised in earlier studies [11].

## References

[1] Barchard KA. Does emotional intelligence assist in the prediction of academic success? Educational and Psychological Measurement. 2003;63(5):840–58. 10.1177/0013164403251333

[2] Augusto-Landa JM, Berrios-Martos MP, López-Zafra E, Aguilar Luzón MC. Relación entre burnout e inteligencia emocional y su impacto en salud mental, bienestar y satisfacción laboral en profesionales de enfermería [Relationship between burnout and emotional intelligence and its impact on mental health, well-being and job satisfaction in nursing professionals]. Ansiedad y Estrés. 2006;12(2):479–93.

[3] Extremera N, Salguero JM, Fernández-Berrocal P. Trait meta-mood and subjective happiness: A 7-week prospective study. Journal of Happiness Studies. 2011;12(3):509–17. 10.1007/s10902-010-9233-7

[4] Bar-On R. The development of a concept of psychological well-being. Unpublished doctoral dissertation. South Africa: Rhodes University; 1988.

[5] Fernández-Berrocal P, Extremera N. La inteligencia emocional y el estudio de la felicidad [Emotional intelligence and the study of happiness]. Revista Interuniversitaria de Formación del Profesorado. 2009;66:85–108.

[6] Goleman D. La inteligencia emocional [Emotional intelligence]. Buenos Aires: Editor Javier Vergara; 1996.

[7] Goleman D. La práctica de la inteligencia emocional [The practice of emotional intelligence]. Barcelona: Kairós; 1999.

[8] Ciarrochi J, Deane FP, Anderson S. Emotional intelligence moderates the relationship between stress and mental health. Personality and Individual Differences. 2002;32(2):197–209. 10.1016/S0191-8869(01)00012-5

[9] Salovey P, Mayer JD. Emotional intelligence. Imagination, cognition and personality. 1990;9(3):185–211. 10.2190/DUGG-P24E-52WK-6CDG

[10] Bar-On R. The Bar-On model of emotional-social intelligence (ESI). Psicothema. 2006;18:13–25. 17295953

[11] Flórez J, Garvía B, Fernández-Olaria R. Síndrome de Down: Neurobiología, Neuropsicología, Salud Mental. Santander: Fundación Iberoamericana Down21, CEPE; 2015.

[12] Ruiz E. Todo un mundo de emociones. Educación emocional y bienestar en el síndrome de Down [A whole world of emotions. Emotional education and well-being in Down syndrome]. Madrid: Editorial CEPE; 2016.

[13] Cebula KR, Moore DG, Wishart JG. Social cognition in children with Down's syndrome: challenges to research and theory building. Journal of Intellectual Disability Research. 2010;54(2):113–34. 10.1111/j.1365-2788.2009.01215.x 19874447

[14] Channell MM, Conners FA, Barth JM. Emotion knowledge in children and adolescents with Down syndrome: A new methodological approach. American Journal on Intellectual and Developmental Disabilities. 2014;119(5):405–21. 10.1352/1944-7558-119.5.405 25148055

[15] Izard CE, Woodburn EM, Finlon KJ, Krauthamer-Ewing ES, Grossman SR, Seidenfeld A. Emotion knowledge, emotion utilization, and emotion regulation. Emotion Review. 2011;3(1):44–52. 10.1177/1754073910380972

[16] Morgan JK, Izard CE, King KA. Construct validity of the Emotion Matching Task: Preliminary evidence for convergent and criterion validity of a new emotion knowledge measure for young children. Social Development. 2010;19(1):52–70. 10.1111/j.1467-9507.2008.00529.x 20376197 PMC2850106

[17] Roper RJ, Reeves RH. Understanding the basis for Down syndrome phenotypes. PLoS Genetics. 2006;2(3). 10.1371/journal.pgen.0020050 PMC1420680 16596169

[18] Potier MC, Rivals I, Mercier G, Ettwiller L, Moldrich R, Laffaire J, et al Transcriptional disruptions in Down syndrome: a case study in the Ts1Cje mouse cerebellum during post‐natal development. Journal of Neurochemistry. 2006;97:104–9. 10.1111/j.1471-4159.2005.03624.x 16635258

[19] Robles-Bello MA, Valencia N, Barba-Colmenero F, Sánchez-Teruel D. Evaluación del perfil cognitivo y de conducta en niños de un centro de atención y desarrollo infantil temprano [Assessment of the cognitive and behavioral profile in children of an early childhood intervention and development center]. Revista Argentina de Clínica Psicológica. 2017;26(3):313–23. 10.24205/03276716.2017.1023

[20] Carr J, Carr JH. Down's syndrome: Children growing up: Cambridge University Press; 1995.

[21] Cunningham C. Families of children with Down syndrome. Down Syndrome Research and Practice. 1996;4(3):87–95.

[22] Sánchez-Teruel D, Robles-Bello MA. Respuesta a un programa de resiliencia aplicado a padres de niños con Síndrome de Down [Responding to a program of resilience applied parents of children with Down Syndrome]. Universitas Psychologica. 2015;14(2):645–57. 10.11144/Javeriana.upsy14-1.rpra

[23] Van Riper M, Cohen WI. Caring for children with Down syndrome and their families. Journal of Pediatric Health Care. 2001;15(3):123–31. 10.1067/mph.2001.110627 11353361

[24] Fernández-Berrocal P, Extremera N. El papel de la Inteligencia Emocional en el alumnado: Evidencias empíricas [The role of Emotional Intelligence in students: Empirical evidence]. Revista Electrónica de Investigación Educativa. 2008;6(2):2004.

[25] Saklofske DH, Austin EJ, Minski PS. Factor structure and validity of a trait emotional intelligence measure. Personality and Individual Differences. 2003;34(4):707–21. 10.1016/S0191-8869(02)00056-9

[26] Schutte NS, Malouff JM, Thorsteinsson EB, Bhullar N, Rooke SE. A meta-analytic investigation of the relationship between emotional intelligence and health. Personality and Individual Differences. 2007;42(6):921–33. 10.1016/j.paid.2006.09.003

[27] Austin EJ, Saklofske DH, Egan V. Personality, well-being and health correlates of trait emotional intelligence. Personality and Individual Differences. 2005;38(3):547–58. 10.1016/j.paid.2004.05.009

[28] Gohm CL, Corser GC, Dalsky DJ. Emotional intelligence under stress: Useful, unnecessary, or irrelevant? Personality and Individual Differences. 2005;39(6):1017–28. 10.1016/j.paid.2005.03.018

[29] Piñar-Chelso MJ, Fernández-Castro J. A new scale to evaluate disruptive passenger management by cabin crew. Aviation Psychology and Applied Human Factors. 2011 10.1027/2192-0923/a00009

[30] Tsaousis I, Nikolaou I. Exploring the relationship of emotional intelligence with physical and psychological health functioning. Stress and Health: Journal of the International Society for the Investigation of Stress. 2005;21(2):77–86. 10.1002/smi.1042

[31] Fernandez-Berrocal P, Alcaide R, Extremera N, Pizarro D. The role of emotional intelligence in anxiety and depression among adolescents. Individual Differences Research. 2006;4(1).

[32] White SW, Oswald D, Ollendick T, Scahill L. Anxiety in children and adolescents with autism spectrum disorders. Clinical Psychology Review. 2009;29(3):216–29. 10.1016/j.cpr.2009.01.003 19223098 PMC2692135

[33] Mazzone L, Ruta L, Reale L. Psychiatric comorbidities in asperger syndrome and high functioning autism: diagnostic challenges. Annals of General Psychiatry. 2012;11(1):16 10.1186/1744-859X-11-16 22731684 PMC3416662

[34] Couzens D, Cuskelly M, Haynes M. Cognitive development and Down syndrome: Age-related change on the Stanford-Binet Test. American Journal on Intellectual and Developmental Disabilities. 2011;116(3):181–204. 10.1352/1944-7558-116.3.181 21591843

[35] Couzens D, Haynes M, Cuskelly M. Individual and environmental characteristics associated with cognitive development in Down syndrome: A longitudinal study. Journal of Applied Research in Intellectual Disabilities. 2012;25(5):396–413. 10.1111/j.1468-3148.2011.00673.x 22890941

[36] Cleland J, Wood S, Hardcastle W, Wishart J, Timmins C. Relationship between speech, oromotor, language and cognitive abilities in children with Down's syndrome. International Journal of Language & Communication Disorders. 2010;45(1):83–95. 10.3109/13682820902745453 19821789

[37] Lanfranchi S, Baddeley A, Gathercole S, Vianello R. Working memory in Down syndrome: is there a dual task deficit? Journal of Intellectual Disability Research. 2012;56(2):157–66. 10.1111/j.1365-2788.2011.01444.x 21726323

[38] Næss K-AB, Melby-Lervåg M, Hulme C, Lyster S-AH. Reading skills in children with Down syndrome: A meta-analytic review. Research in Developmental disabilities. 2012;33(2):737–47. 10.1016/j.ridd.2011.09.019 22115916

[39] Stern JA, Gadgil MS, Blakeley-Smith A, Reaven JA, Hepburn SL. Psychometric properties of the SCARED in youth with autism spectrum disorder. Research in Autism Spectrum Disorders. 2014;8(9):1225–34. 10.1016/j.rasd.2014.06.008 25147580 PMC4136967

[40] Herrera L, Buitrago RE, Lorenzo O, Badea M. Socio-Emotional Intelligence in Colombian Children of Primary Education. An analysis in rural and urban settings. Procedia-Social and Behavioral Sciences. 2015;203:4–10. 10.1016/j.sbspro.2015.08.251

[41] Bar-On R, Parker JDA. The Bar-On Emotional Quotient Inventory: Youth version (EQ-i:YV) technical manual. Toronto, Canada: Multi-Health system, Incorporated; 2000.

[42] Esnaola I, Revuelta L, Ros I, Sarasa M. The development of emotional intelligence in adolescence. Annals of Psychology. 2017;33(2):327–33. 10.6018/analesps.33.2.251831

[43] Mason-Apps E, Stojanovik V, Houston-Price C, Buckley S. Longitudinal predictors of early language in infants with Down syndrome: A preliminary study. Research in Developmental Disabilities. 2018;81:37–51. 10.1016/j.ridd.2017.12.021 29329955

[44] Pujol J, del Hoyo L, Blanco-Hinojo L, de Sola S, Macià D, Martínez-Vilavella G, et al Anomalous brain functional connectivity contributing to poor adaptive behavior in Down syndrome. Cortex. 2015;64:148–56. 10.1016/j.cortex.2014.10.012 25461715

[45] Pochon R, Touchet C, Ibernon L. Emotion recognition in adolescents with Down syndrome: a nonverbal approach. Brain Sciences. 2017;7(6):55 10.3390/brainsci7060055 28545237 PMC5483628

[46] Moore DG. Reassessing emotion recognition performance in people with mental retardation: A review. American Journal on Mental Retardation. 2001;106(6):481–502. 10.1352/0895-8017(2001)106<0481:RERPIP>2.0.CO;2 11708935

[47] Abbeduto L, Warren SF, Conners FA. Language development in Down syndrome: From the prelinguistic period to the acquisition of literacy. Mental Retardation and Developmental Disabilities Research Reviews. 2007;13(3):247–61. 10.1002/mrdd.20158 17910087

[48] Mundy P, Kasari C, Sigman M, Ruskin E. Nonverbal communication and early language acquisition in children with Down syndrome and in normally developing children. Journal of Speech, Language, and Hearing Research. 1995;38(1):157–67. 10.1044/jshr.3801.157 7537345

[49] Cebula KR, Wishart JG, Willis DS, Pitcairn TK. Emotion recognition in children with Down Syndrome: influence of emotion label and expression intensity. American Journal on Intellectual and Developmental Disabilities. 2017;122(2):138–55. 10.1352/1944-7558-122.2.138 28257244

[50] Hippolyte L, Barisnikov K, Van der Linden M, Detraux J-J. From facial emotional recognition abilities to emotional attribution: A study in Down syndrome. Research in Developmental Disabilities. 2009;30(5):1007–22. 10.1016/j.ridd.2009.02.004 19297130

[51] Carvajal F, Fernández-Alcaraz C, Rueda M, Sarrión L. Processing of facial expressions of emotions by adults with Down syndrome and moderate intellectual disability. Research in Developmental Disabilities. 2012;33(3):783–90. 10.1016/j.ridd.2011.12.004 22240141

[52] García Alonso I, Medina Gómez M. Comportamiento, lenguaje y cognición de algunos síndromes que cursan con discapacidad intelectual [Behavior, language and cognition of some syndromes with intellectual disability]. INFAD Revista de Psicología, International Journal of Developmental and Educational Psychology 2017, N 1, v 4, p 55–66. 2017.

[53] White SW, Oswald D, Ollendick T, Scahill L. Anxiety in children and adolescents with autism spectrum disorders. Clinical Psychology Review. 2009;29(3):216–29. Epub 2009/02/19. 10.1016/j.cpr.2009.01.003 PubMed Central PMCID: PMC2692135. 19223098 PMC2692135

[54] Ferrándiz C, Hernández D, Bermejo R, Ferrando M, Sáinz M. Social and emotional intelligence in childhood and adolescence: Spanish validation of a measurement instrument. Revista de Psicodidáctica. 2012;17(2):309–39.

[55] Bar-On R. How important is it to educate people to be emotionally and socially intelligent, and can it be done? Perspectives in Education. 2003;21(4):3–15.

[56] Bar-On R. The Bar-On Emotional Quotient Inventory (EQ-i): Rationale, description and summary of psychometric properties. In: Geher G, editor. Hauppauge, NY: Nova Science Publishers; 2004 p. 111–42.

[57] García-Ros R, Pérez-González F. Validez predictiva e incremental de las habilidades de autorregulación sobre el éxito académico en la universidad [Predictive and incremental validity of self-regulation skills on academic success in college]. Revista de Psicodidáctica. 2011;16(2):231–50.

[58] Bar-On R, Handley R, Fund S. The impact of emotional intelligence on performance. In: Vanessa Druskat FS, & Gerald Mount, editor. Linking emotional intelligence and performance at work: Current research evidence with individuals and groups. Mahwah, NJ: Lawrence Erlbaum; 2006 p. 3–19.

[59] Ferrando AA, Paddon-Jones D, Hays NP, Kortebein P, Ronsen O, Williams RH, et al EAA supplementation to increase nitrogen intake improves muscle function during bed rest in the elderly. Clinical Nutrition. 2010;29(1):18–23. 10.1016/j.clnu.2009.03.009 19419806

[60] Limonero JT, Tomás-Sábado J, Fernández-Castro J. Perceived emotional intelligence and its relation to tobacco and cannabis use among university students. Psicothema. 2006;18:95–100. 17295964

[61] Mestre JM, Guil R, Lopes PN, Salovey P, Gil-Olarte P. Emotional intelligence and social and academic adaptation to school. Psicothema. 2006;18:112–7. 17295967

[62] Kun B, Urbán R, Paksi B, Csóbor LV, Oláh A, Demetrovics Z. Psychometric characteristics of the Emotional Quotient Inventory, Youth Version, Short Form, in Hungarian high school students. Psychological Assessment. 2012;24(2):518 10.1037/a0026013 22004539

[63] Stanimirovic R, Hanrahan S. Examining the dimensional structure and factorial validity of the Bar-On Emotional Quotient Inventory in a sample of male athletes. Psychology of Sport and Exercise. 2012;13(1):44–50. 10.1016/j.psychsport.2011.07.009

[64] Parker AR, Sieber OM, Shi C, Hua L, Takao M, Tomlinson IP, et al Cells with pathogenic biallelic mutations in the human MUTYH gene are defective in DNA damage binding and repair. Carcinogenesis. 2005;26(11):2010–8. 10.1093/carcin/bgi166 15987719

[65] El Hassan K, El Sader M. Adapting and validating the BarOn EQ–i: YV in the Lebanese context. International Journal of Testing. 2005;5(3):301–17. 10.1207/s15327574ijt0503_7.

[66] Robles-Bello MA, Cubero T, Muela JA, Montes-Berges B. Inteligencia Emocional en síndrome de Down. Libro de Actas Avances en el estudio de la motivación y de la emoción [Emotional Intelligence in Down syndrome. Book of Proceedings Advances in the study of motivation and emotion]. VIII Simposio de la Asociación de Motivación y Emoción; Granada: Universidad de Granada; 2014.

[67] Marchal S, Marx I, Van Mechelen N. Minimum income protection in the austerity tide. IZA Journal of European Labor Studies. 2016;5(1):4.

[68] Down Spain Map of our associations in Spain Santander: Fundación Iberoamericana Down21, CEPE; 2018 [updated 9 may 2019]. Available from: https://www.sindromedown.net/conocenos/nuestras-entidades/.

[69] Schalock RL, Borthwick-Duffy SA, Bradley VJ, Buntinx WH, Coulter DL, Craig EM, et al Intellectual disability: Definition, classification, and systems of supports: ERIC; 2010.

[70] Kaufman AS. K-BIT, Test Breve de Inteligencia de KAUFMAN [K-BIT, Brief Intelligence Test of KAUFMAN]. Madrid: Pearson; 1997.

[71] Graham JW. Missing data: Analysis and design: Springer Science & Business Media; 2012.

[72] Lorenzo-Seva U, Ferrando PJ. FACTOR: A computer program to fit the exploratory factor analysis model. Behavior Research Methods. 2006;38(1):88–91. 10.3758/bf03192753 16817517

[73] Baglin J. Improving your exploratory factor analysis for ordinal data: A demonstration using FACTOR. Practical Assessment, Research, and Evaluation [Internet]. 2014; 19(1):[5 p.]. Available from: http://pareonline.net/getvn.asp?v=19&n=5.

[74] Timmerman ME, Lorenzo-Seva U. Dimensionality assessment of ordered polytomous items with parallel analysis. Psychological Methods. 2011;16(2):209 10.1037/a0023353 21500916

[75] Garrido LE, Abad FJ, Ponsoda V. A new look at Horn’s parallel analysis with ordinal variables. Psychological Methods. 2013;18(4):454 10.1037/a0030005 23046000

[76] Jennrich RI. Admissible values of γ in direct oblimin rotation. Psychometrika. 1979;44(2):173–7. 10.1007/BF02293969

[77] Horn JL. A rationale and test for the number of factors in factor analysis. Psychometrika. 1965;30(2):179–85. 14306381 10.1007/BF02289447

[78] O’Connor BP. SPSS and SAS programs for determining the number of components using parallel analysis and Velicer’s MAP test. Behavior research methods, instruments, & computers. 2000;32(3):396–402. 10.3758/bf03200807 11029811

[79] Glorfeld LW. An improvement on Horn's parallel analysis methodology for selecting the correct number of factors to retain. Educational and Psychological Measurement. 1995;55(3):377–93.

[80] Rodríguez-Ayán MN, Ruiz-Díaz MA. Atenuación de la asimetría y de la curtosis de las puntuaciones observadas mediante transformaciones de variables: Incidencia sobre la estructura factorial [Attenuation of the asymmetry and kurtosis of the observed scores through transformations of variables: Incidence on the factorial structure]. Psicológica: Revista de Metodología y Psicología Experimental 2008;29(2):205–27.

[81] Kline RB. Principles and practice of structural equation modeling. New York: The Guilford Press; 2015.

[82] Lloret-Segura S, Ferreres-Traver A, Hernández-Baeza A, Tomás-Marco I. El análisis factorial exploratorio de los ítems: una guía práctica, revisada y actualizada [Exploratory Item Factor Analysis: a practical guide revised and updated]. Anales de Psicología/Annals of Psychology. 2014;30(3):1151–69. 10.6018/analesps.30.3.199361

[83] Erhart M, Hagquist C, Auquier P, Rajmil L, Power M, Ravens‐Sieberer U, et al A comparison of Rasch item‐fit and Cronbach's alpha item reduction analysis for the development of a Quality of Life scale for children and adolescents. Child: Care, Health and Development 2010;36(4):473–84. 10.1111/j.1365-2214.2009.00998.x 19702637

[84] Zijlmans EA, Tijmstra J, van der Ark LA, Sijtsma K. Item-score reliability as a selection tool in test construction. Frontiers in Psychology. 2019;9:2298 10.3389/fpsyg.2018.02298 30687144 PMC6336834

[85] Mardia KV. Measures of multivariate skewness and kurtosis with applications. Biometrika. 1970;57(3):519–30. 10.1093/biomet/57.3.519

[86] Morales G, López O. El síndrome de down y su mundo emocional [Down syndrome and its emotional world]. Sevilla: Trillas; 2007.

[87] Ruiz E, Álvarez R, Arce A, Palazuelos I, Schelstraete G. Programa de educación emocional. Aplicación práctica en niños con síndrome de Down [Emotional education program. Practical application in children with Down syndrome]. Revista Síndrome de Down. 2009;26(103):126–39.

[88] Down-Spain. Emociona-Down. Programa de educación emocional. Guía de orientaciones didácticas para mediadores emocionales [Emotion-Down. Emotional education program. Educational guidance guide for emotional mediators] Madrid: Down Spain and Gmp Foundation; 2017. Available from: http://www.sindromedown.net/wp-content/uploads/2018/01/Programa-Emociones.-Gui%CC%81a-del-mediador.pdf.

[89] Kasari C, Freeman SF, Hughes MA. Emotion recognition by children with Down syndrome. American Journal on Mental Retardation. 2001;106(1):59–72. 10.1352/0895-8017(2001)106<0059:ERBCWD>2.0.CO;2 11246714

[90] Bar-On R. EQ-i, Bar-On Emotional Quotient Inventory: A measure of emotional intelligence. (Technical manual). Toronto, Canadá: Multi-Health Systems; 1997.

[91] Shi D, DiStefano C, McDaniel HL, Jiang Z. Examining chi-square test statistics under conditions of large model size and ordinal data. Structural Equation Modeling: A Multidisciplinary Journal. 2018;25(6):924–45. 10.1080/10705511.2018.1449653

[92] Klein AM, Houtkamp EO, Salemink E, Baartmans JM, Rinck M, van der Molen MJ. Differences between self-and peer-rated likability in relation to social anxiety and depression in adolescents with mild intellectual disabilities. Research in Developmental Disabilities. 2018;80:44–51. 10.1016/j.ridd.2018.05.016 29908392

[93] García-Alba J, Ramos-Alla JF, Martín-Palacios ME. Variabilidad del perfil cognitivo en escolares y adultos con Síndrome de Down [Variability cognitive profile in children and adults with Down Syndrome]. Revista INFAD de Psicología International Journal of Developmental and Educational Psychology. 2014;3(1):203–12.

